# Thyroid function in the etiology of fatigue in breast cancer

**DOI:** 10.18632/oncotarget.25438

**Published:** 2018-05-22

**Authors:** Nagi B. Kumar, Angelina Fink, Silvina Levis, Ping Xu, Roy Tamura, Jeffrey Krischer

**Affiliations:** ^1^ H. Lee Moffitt Cancer Center & Research Institute, Tampa, FL 33612, USA; ^2^ Geriatric Research, Education and Clinical Center, Miami Veterans Affairs Medical Center, Miami, FL 33125, USA; ^3^ Pediatrics Epidemiology Center at University of South Florida, Health Informatics Institute, Tampa, FL 33612, USA

**Keywords:** thyroid function, fatigue, hypothyroidism, subclinical hypothyroidism, breast cancer

## Abstract

**Background:**

Cancer related fatigue (CRF), reported in about 90% of breast cancer patients receiving chemotherapy, and has a profound impact on physical function, psychological distress and quality of life. Although several etiological factors such as anemia, depression, chronic inflammation, neurological pathology and alterations in metabolism have been proposed, the mechanisms of CRF are largely unknown.

**Methods:**

We conducted a pilot, prospective, case-control study to estimate the magnitude of change in thyroid function in breast cancer patients from baseline to 24 months, compared to cancer-free, age-matched controls. Secondary objectives were to correlate changes in thyroid function and obesity over time with fatigue symptoms scores in this patient population.

**Results:**

The proportion of women with breast cancer who developed subclinical or overt hypothyroidism (TSH >4.0 mIU/L) from baseline to year 1 was significantly greater compared to controls (9.6% vs. 5%; p=0.02). Subjects with breast cancer reported significantly worse fatigue symptoms than age-matched controls, as indicated by higher disruption indices (p<0.001 at baseline, p=0.02 at year 1, p=0.09 at year 2). Additionally, a significant interaction effect on disruption index score (p=0.019), general level of activity over time (p=0.006) and normal work activity (p= 0.002) was observed in the subgroup of women with BMI>30.

**Conclusion:**

Screening breast cancer patients for thyroid function status at baseline and serially post-treatment to evaluate the need for thyroid hormone replacement may provide for a novel strategy for treating chemotherapy-induced fatigue.

## INTRODUCTION

In 2017, it is estimated that there will be 252,710 new cases of female breast cancer and an estimated 40,610 women will die of this disease in the United States (US) [1]. Due to significant advances in early detection and treatment, it is estimated that there are over 3.5 million breast cancer survivors in the US, dramatically increasing the number of cancer-affected life-years in the US. Breast cancer survivors report symptoms of fatigue, weight gain, pain, poor sleep, cognitive impairment and depression that persists post treatment. Of these symptoms, persistent cancer related fatigue (CRF) has been reported as one of the most distressing long-term side-effects of cancer treatment [2–5]. CRF is reported in about 90% of breast cancer patients receiving radiation and chemotherapy [6–13], regardless of whether the cancer is invasive [8], with residual fatigue often persisting for as long as 10 years after the completion of treatments [14–16]. In contrast, there is little evidence that patients who receive only regional therapy (i.e., surgery plus adjuvant radiotherapy) experience clinically significant fatigue as a long-term treatment side effect [17]. Compared with fatigue experienced by healthy individuals, CRF is accompanied by loss of drive, lack of energy, depressive mood and loss of vigor and vitality. CRF is disproportionate to the level of exertion, disabling, interferes with usual functioning [1], and is less likely to be relieved with rest and sleep [18, 19]. Because of its significant effect on physical function and ability to perform activities of daily living, resulting in psychological distress, the impact of CRF on a cancer patient's quality of life (QoL) is profound [20–22]. Fatigue has also been reported to have an impact on the patient’s ability to work and consequently may add to the financial burden of the cancer patient. These effects can extend to caregivers and family members, who may also have to reduce their working capacity in order to provide additional care for a patient with CRF [23]. CRF and related symptoms can thus affect the QoL during cancer treatment as well as survival, with effects on productivity, family functioning, as well as both physiological and psychological comorbidity. Additionally, significant individual variability in its clinical expression, determinants and sequela [24] have been observed, thus complicating approaches for management of fatigue in this population.

Although several etiological factors such as anemia, depression [3, 4], chronic inflammation, neurological pathology [25] and alterations in metabolism [26] have been proposed, the mechanisms of CRF are largely unknown. In a prior prospective study of pre-menopausal and post-menopausal stage I-IIIB breast cancer patients receiving adjuvant chemotherapy, we observed progressive increase in symptoms of fatigue, concurrent weight gain, amenorrhea, lowered physical activity or lethargy- symptoms also common to hypothyroidism. In addition, 20% of the breast cancer patients screened were already diagnosed with overt clinical hypothyroidism at diagnosis, prior to start of antineoplastic agents or radiation therapy and were not included [27]. Although the pathophysiology of thyroid toxicity induced by antineoplastic agents is not fully clarified [28], the high incidence of hypothyroidism observed in this patient population is significant, compared to the 3.7% observed in the American population [29]. More recently, thyroid dysfunction such as primary hypothyroidism has been reported to be a common side effect in 20-50% of cancer patients receiving targeted therapies such as tyrosine-kinase inhibitors, bexarotene, radio-iodine-based cancer therapies, denileukin diftitox, alemtuzumab, interferon-α, interleuin-2, ipilimumab, tremelimumab and lenalidomide [30, 31]. Other patient populations reporting significant loss of thyroid function are patients receiving radiation for the treatment of brain tumors [32, 33]. Additionally, symptoms of overt and subclinical hypothyroidism observed during treatment may result in reduction in dose or discontinuation of therapy [28, 31], with significant impact on cancer treatment outcomes. Other symptoms observed with hypothyroidism during treatment include cardiovascular mortality [30, 34] progressive weight gain [7, 13, 35], weakness, hypothermia, depression and lower health related quality of life. Over the past 2 decades, we and others have reported that adult weight gain, obesity, body composition and body fat distribution are risk factors for breast cancer and prognosis [27]. A major factor that may contribute to weight gain in this population during treatment is the reported lethargy and inactivity or decreased physical activities that have been related to fatigue. Based on this early evidence, several pilot and feasibility studies have been undertaken to examine interventions with physical activity and obesity management to reduce fatigue related symptoms and improve quality of life [36–39]. However, to-date, no studies have examined the association of markers of thyroid dysfunction in the etiology of fatigue in breast cancer patients. Both subclinical and overt hypothyroidisms are treatable conditions. Thus, if thyroid dysfunction prior to and after treatment were to be demonstrated as putative, this would represent a new avenue for intervention, in addition to programs to promote weight management and physical activity, to prevent or manage fatigue and related symptoms in women post-treatment for breast cancer. Based on our observations and those of others, we hypothesize that CRF in breast cancer patients may have a physiological etiology and may be associated to decreasing thyroid function post-treatment with antineoplastic agents. Our objective was to conduct a pilot, prospective, case-control study to estimate the proportion of subjects with hypothyroidism in newly diagnosed breast cancer patients (prior to chemotherapy) compared to cancer-free, age matched controls and to estimate the magnitude of change in thyroid function in breast cancer patients from baseline to 24 months, compared to cancer-free, age-matched controls. Secondary objectives were to correlate variation in markers of obesity over time with fatigue symptoms scores in this patient population.

## RESULTS

**Figure 1:**
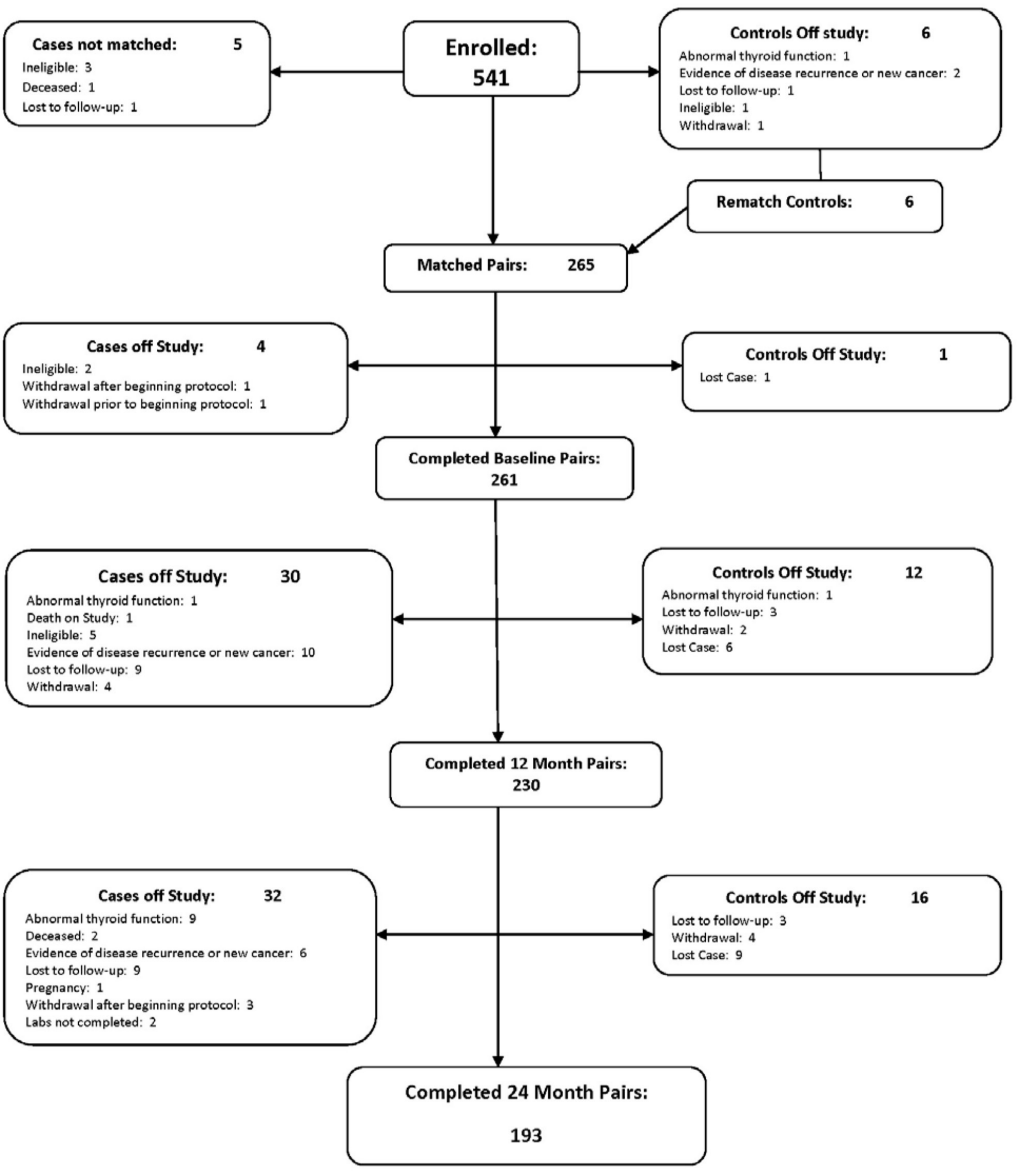
Thyroid function in breast cancer patients on chemotherapy: consort diagram

A total of 261 age-matched pairs were enrolled, with 230 pairs completing 12 months and 193 pairs completing 24 months of observation. The consort diagram (Figure 1) demonstrates the number of case control pairs recruited and followed at 12 and 24 months, including attrition and reason for attrition. A significantly larger number of cases went off study (64 cases) compared to 29 controls. Sixteen out of 64 cases met off study criteria due to disease recurrence or new cancer diagnosis. Additionally, the proportion of women who had subclinical or overt hypothyroidism at baseline was not different between cases and controls. Demographic and clinical characteristics of participants (breast cancer cases vs. controls) at baseline (n=261 matched evaluable pairs) appears in Table 1.

**Table 1:** Demographic and clinical characteristics of participants (breast cancer cases vs. controls) at baseline

Characteristics Number of participants	Breast cancer cases (n=261)	Age-matched controls (n=261)	P value^*^
Age, years, mean (SD)^*^	50.65 (10.59)	50.38 (10.37)	0.11
BMI, kg/m^2^, mean (SD))	29.10 (6.50)	28.43 (6.36)	0.22
BMI categories, n (%)			0.63
< 25	80 (30.65)	88 (33.72)	
25-30	81 (31.03)	82 (31.42)	
>30	100 (38.31)	91 (34.87)	
Race, n (%)			0.02
Black	40 (15.33%)	20 (7.66%)	
White	212 (81.23%)	232 (88.89%)	
Other or Unknown	9 (3.45%)	9 (3.45%)	
Ethnicity, n (%)			0.75
Hispanic or Latino	9 (3.45%)	8 (3.07%)	
Not Hispanic or Latino	249 (95.40%)	248 (95.02%)	
Unknown	3 (1.15%)	5 (1.92%)	
History of benign breast disease, n (%)			<0.001
Yes	60 (22.99)	80 (30.65)	
No	133 (50.96)	181 (69.35)	
Unknown	68 (26.05)	0	

^*^SD: Standard Deviation.

P value: Paired T test was used to compare continuous variables between cases and controls; Cochran-Mantel-Haenszel test was used to compare categorical variables.

Although subjects were well matched in age and community, a significantly greater number of women were Black in the case arm compared to controls (p =0.02). Subjects in the control arm had a significantly higher incidence of benign breast disease compared to cases (p =<0.01). Table 2A summarizes the data distribution of thyroid function classes based on serum TSH between cases and controls at baseline, year 1 and year 2. The proportion of women who had subclinical or overt hypothyroidism (TSH >4.0 mIU/L) at baseline was not different between cases and controls (p=0.41). However, a greater number of women with breast cancer developed subclinical or overt hypothyroidism compared to controls at year 1 (p=0.02). Although more cases with subclinical or overt hypothyroidism were identified in year 2 compared to controls, this number was not statistically significant (p=0.20). Table 2B summarizes the distribution serum Free thyroxine (FT4) fraction between cases and controls at baseline, year 1 and year 2. Although the baseline FT4 concentrations did not differ between cases and controls, a greater number of breast cancer cases had a decrease in serum concentrations of FT4 at year 1 (p=o.03). Although FT4 continued to decrease in cases compared to controls, these decreases were not statistically significant at 2 years (p-0.06). However, overall, FT4 remained within normal ranges for this age group of patients. Table 2C provides the comparative summary of Thyroid Peroxidase Antibody (TPO Ab) in Breast Cancer cases and controls. Although more cases were positive for TPOAb at 1 year compared to controls, these differences were not statistically significant.

**Table 2A:** Frequency of thyroid function in breast cancer cases and controls at baseline, year 1 and year 2

(N: observed pairs with TSH data)	Baseline (N=261)			Year 1 (N=223)			Year 2 (N=193)		
TSH range mIU/L	Cases	Controls	P value	Cases	Controls	P value	Cases	Controls	P vaue
	n (%)	n (%)		n (%)	n (%)		n (%)	n (%)	
**Normal thyroids:** (TSH<=4.0)	246(94.2)	250(95.8)	0.41	202(90.6)	214(96.0)	0.02	178 (92.3)	184(95.3)	0.2
**Subclinical hypothyroidism** (TSH > 4.0 and <=10.0)	15 (5.8)	10 (3.8)		16 (7.2)	9 (4.0)		13(6.7)	9 (4.7)	
**Overt Hypothyroidism:** (TSH >10.0)	0 (0.0)	1 (0.4)		5 (2.2)	0 (0.0)		2 (1.0)	0 (0.0)	

P value: McNemar test was used to compare if the proportions of TSH concentrations differed between cases and controls.

**Table 2B:** Comparative summary of free thyroxine (free T4) fraction in breast cancer cases and controls

Free T4 (ng/dL) (# of observed pairs with Free T4 data)	Cases	Controls	P value
	median (Q1-Q3)	median (Q1-Q3)	
Baseline (n=259)	1.10 (0.99-1.22)	1.08 (1.00-1.17)	0.24
Year 1 (n=225)	1.08 (0.98-1.19)	1.10 (1.02-1.22)	**0.03**
Year 2 (n=188)	1.10 (1.01-1.20)	1.11 (1.02-1.23)	0.06

P value: Wilcoxon signed rank test was used to compare Free T4 between groups at each visit.

**Table 2C:** Comparative summary of thyroid peroxidase antibody (TPO Ab) in breast cancer cases and controls

(N: observed pairs with TPO Ab data)	Baseline (N=260)			Year 1 (N=224)			Year 2 (N=189)		
TPO Autoantibody	Cases	Controls	P value	Cases	Controls	P value	Cases	Controls	P value
	n (%)	n (%)		n (%)	n (%)		n (%)	n (%)	
Ab negative < 35.0 IU/mL	236 (90.8)	239 (91.9)	0.62	201 (89.7)	208 (92.9)	0.24	172 (91.0)	173 (91.5)	0.85
Ab positive >=35.0 IU/mL	24 (9.2)	21 (8.0)		23 (10.3)	16 (7.1)		17 (9.0)	16 (8.5)	

P value: McNemar test was used to compare if the proportions of TPO autoantibody positivity differed between cases and controls.

Fatigue symptoms in breast cancer cases and controls are summarized in Table 3. As expected, subjects with breast cancer reported significantly greater fatigue symptoms than age-matched controls. The disruption index for subjects with breast cancer was significantly higher than controls at baseline and year 1 (p<0.001 at baseline, p=0.02 at year 1). Although the difference of disruption index was smaller between cases and controls, cases still had a higher scores than controls and the statistical significance remained at year 2 (p=0.04).

**Table 3:** Summary of fatigue symptoms in breast cancer cases and controls

Fatigue Symptoms (Number of pairs with FSI)	Baseline (N=261)			Year 1 (N=227)			Year 2 (N=191)		
	Cases	Controls	P value	Cases	Controls	P value	Cases	Controls	P value
	Median (Q1-Q3)	Median (Q1-Q3)		Median (Q1-Q3)	Median (Q1-Q3)		Median (Q1-Q3)	Median (Q1-Q3)	
1. most fatigued during the past week	4 (2 - 6)	5 (3- 6)	0.43	4 (2- 7)	5 (2- 7)	0.73	5 (2- 7)	5 (2- 6)	0.95
2. least fatigued during the past week	1 (0- 3)	1 (0- 2)	**0.001**	1.5 (0 - 3)	1 (0 - 2)	**0.01**	1.5 (0- 3)	1 (0- 2)	0.07
3. fatigue on the average during the past week	3 (1- 4)	2 (1 - 4)	**0.03**	3 (1- 5)	3 (1 – 4)	0.10	3 (1- 4)	3 (1- 4)	0.58
4. fatigue right now	2 (0 – 4)	1 (0 - 3)	**<0.001**	2 (0- 5)	2 (0- 3)	**0.02**	2 (0- 4)	2 (0- 3)	0.14
5. fatigue interfered with your general level of activity	2 (0 – 4)	1 (0 - 2)	**<0.001**	1 (0- 5)	2 (0 – 3)	0.055	2 (0- 4)	1 (0- 3)	**0.01**
6. fatigue interfered with your ability to bathe and dress yourself	0 (0 - 1)	0 (0 - 0)	**<0.001**	0 (0 – 1)	0 (0 - 0)	**<0.001**	0 (0- 0)	0 (0- 0)	**0.01**
7. interfered with your normal work activity	1 (0 - 4)	0 (0 - 2)	**<0.001**	1 (0 - 4)	1 (0- 2)	**<0.001**	1 (0- 3)	1 (0- 2)	**0.001**
8. fatigue interfered with your ability to concentrate	1 (0 - 4)	1 (0 - 2)	**<0.001**	2 (0 - 4)	1 (0- 3)	**0.02**	1 (0- 3)	1 (0- 3)	0.17
9. fatigue interfered with your relations with other people	1 (0 - 3)	1 (0- 2)	**<0.001**	1 (0- 3)	1 (0- 2)	0.08	0 (0- 3)	1 (0- 2)	0.10
10. fatigue interfered with your enjoyment of life	1 (0- 4)	1 (0- 2)	**<0.001**	1 (0- 4)	1 (0- 2)	**0.01**	1 (0 - 3)	1 (0- 2)	0.12
11. fatigue interfered with your mood	2 (0 - 4)	1 (0- 2)	**<0.001**	1 (0- 4)	1 (0- 3)	**0.03**	1(0- 4)	1 (0- 3)	0.26
12. how many days, in the past week, you felt fatigued	3 (1- 7)	2 (1- 4)	**<0.001**	3 (1 - 7)	2 (1- 4)	**0.002**	3 (1 - 6)	3 (1- 4)	0.06
13. how much of the day, on average	2 (1 - 5)	2 (1- 3)	**<0.001**	3 (1- 5)	2 (1- 3)	**<0.001**	2 (1 - 4)	2 (1- 3)	**0.04**
14. daily pattern of your fatigue	3 (1 - 3)	3 (2- 3)	0.55	3 (2- 3)	3 (2 - 3)	0.60	2 (2- 3)	3 (2- 3)	0.56
Disruption Index (Total sum of items 5-11)	10 (2 - 24)	5 (1-12)	**<0.001**	10 (2 - 24)	7 (1-16)	**0.01**	7 (1- 22)	6 (1-14)	**0.04**

Note: Higher score indicated worse fatigue.

Q1: first quartile; Median: second quartile; Q3: third quartile.

P value: Wilcoxon rank sum test to compare symptom verity between cases and controls at each visit. P value was **NOT** adjusted for multiple comparisons. In bold < 0.05.

**Figure 2:**
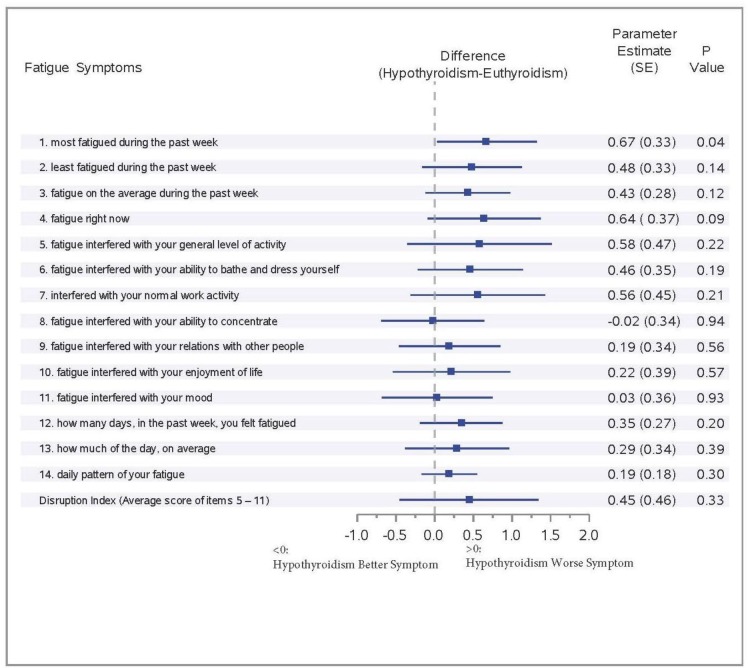
Effect of hypothyroidism on fatigue symptoms among breast cancer patients: forrest plot

Figure 2 illustrates the effect of increasing serum TSH concentration of >4.0 mIU/L (subclinical or overt hypothyroidism) on fatigue symptoms in patients diagnosed with breast cancer using the GEE repeated regression analysis with adjustment for age and BMI. The parameter estimates for hypothyroidism were greater than zero for all fatigue symptoms, except fatigue that interfered with subjects’ ability to concentrate. Increasing concentrations of TSH >4.0 mIU/L were associated with worse symptoms of fatigue in breast cancer patients after chemotherapy. However, only fatigue during the past week achieved statistically significance with hypothyroidism (p=0.04).

Table 4 provides the summary of the fatigue symptoms over time in subgroups stratified by BMI (normal: BMI < 25; overweight: BMI 25-30; obese: BMI >30). A GEE model was used to assess the impact of BMI on the Disruption Index over time. BMI levels had a significant impact on the Disruption Index. Women classified as overweight or obese reported worse fatigue symptoms than those with normal BMI (overweight vs. normal p=0.03; obese vs. normal p=0.02). However, there was no interaction effect of BMI with either group (women with breast cancer vs. health controls, p=0.82) or thyroid function status (hypothyroidism vs. euthyroidism, p=0.92) (As there is no significant interaction, the results were not provided in detail). However, overall, BMI significantly impacted fatigue that interfered with subjects' general level of activity. Obese women in both groups (breast cancer patients and controls) reported significantly worse symptoms than the women with normal BMI (p<0.001). Overweight women also reported worse fatigue that interfered with their general level of activity over time compared to women with normal BMI, but the difference was not statistically significant (p=0.06). Higher BMI also was found to significantly increase fatigue that interfered with subjects' normal work activity (overweight vs. normal: p=0.02, obese vs. normal p=0.01). However, BMI did not show an interaction effect with group (cases vs. controls) or thyroid function status (hypothyroidism vs. euthyroidism) on fatigue that interfered with their normal work activity.

**Table 4:** Fatigue symptoms over time in subgroups stratified by BMI

Median (Q1-Q3)	Disruption Index Summary Score	Fatigue interfered with general level of activity	Fatigue interfered with normal work activity
	**Baseline Visit**		
Normal^*^	6.0 (1.0-13.0)	1.0 (0.0-3.0)	0.0 (0.0-2.0)
Overweight^**^	7.0 (1.0-18.0)	1.0 (0.0-3.0)	1.0 (0.0-3.0)
Obese^***^	7.5 (2.0-18.0)	2.0 (0.0-4.0)	1.0 (0.0-3.0)
	**Year 1 Visit**		
Normal	7.0 (1.0-19.0)	1.0 (0.0-4.0)	1.0 (0.0-2.0)
Overweight	7.0 (2.0-17.0)	1.5 (0.0-4.0)	1.0 (0.0-3.0)
Obese	9.0 (2.0-21.0)	2.0 (0.0-5.0)	1.0 (0.0-3.0)
	**Year 2 Visit**		
Normal	5.0 (1.0-15.0)	1.0(0.0-3.0)	0.0(0.0-2.0)
Overweight	6.0 (1.0-18.0)	1.0 (0.0-4.0)	1.0 (0.0-3.0)
Obese	9.0 (2.0-18.5)	2.0 (0.0-4.0)	1.0 (0.0-3.0)

^*^ Normal BMI < 25 kg/m^2^; Overweight: BMI 25-30 kg/m^2^; BMI >30 kg/m^2^; Q1: first quartile; Median: second quartile; Q3: third quartile.

## DISCUSSION

Although the proportion of women who had subclinical or overt hypothyroidism at baseline was not different between breast cancer cases and controls, our study demonstrated that the proportion of women with breast cancer who developed subclinical or overt hypothyroidism post treatment for breast cancer was significantly greater compared to controls. Subjects with breast cancer reported significantly worse fatigue symptoms than age-matched controls, as indicated by higher disruption index, justifying a need to screen this patient population at start of treatment as well as serially post-treatment to evaluate the need for thyroid hormone replacement and providing for a novel strategy for treating chemotherapy-induced fatigue. Previous studies have examined the association of several cancer and cancer-treatmentrelated biological and psychological contributors to CRF. Although the etiology of CRF maybe multifactorial and involving change in several psychological and physiological biomarkers, the focus of the current pilot study was to examine the role of thyroid dysfunction in breast cancer patients as well as the etiology of CRF in a population of breast cancer patients, post chemotherapy with AC plus a taxane. Since the proportion of women who had subclinical or overt hypothyroidism at baseline was not different between cases and controls, the study failed to indicate the association between thyroid dysfunction and breast cancer itself, but it is more compatible with a possible direct effect of chemotherapy on thyroid gland activity, and/or on thyroid hormone transport and metabolism. In cancer patients, thyroid function is generally thought to be vulnerable to chemotherapy. The ability of chemotherapeutic agents to influence thyroid hormones was previously reported [40]. Previous observations, concerning two chemotherapeutic agents, namely the mitotane and the fluorouracil, indicated that these drugs are capable to alter T4 and T3 transport in serum, by increasing serum TBG concentration [41, 42]. To our knowledge, our study was the first to prospectively observe the association between decreasing thyroid function, as indicated by increasing TSH and decreasing FT4 to increased symptoms of fatigue in patients diagnosed with breast cancer, post chemotherapy. Our study was also the first to identify that women (both cases and controls) with a baseline BMI >30, are a subgroup of women who report significantly greater disruption index scores based on the fatigue symptoms inventory, compared to patients with a BMI <30.

Thyroid hormones play vital role in regulating various metabolic functions in multiple organs. Disruption or change in thyroid hormone axis result in hypothyroidism or hyperthyroidism [34]. Although the classic clinical spectrum of hypothyroidism with symptoms of lethargy and myxedema is well known to the practitioner, it is rarely seen in today’s clinical practice [43]. In contrast, practitioners frequently see patients with “subclinical hypothyroidism” or “mild hypothyroidism” [9, 43]. Additionally, because of the large variation in clinical presentation and general absence of symptom specificity, the definition of hypothyroidism is predominantly biochemical. Primary hypothyroidism is defined by TSH concentrations above the reference range (>4.0 mIU/L) and free thyroxine concentrations below the reference range, which is dependent on the type of assay used and the population studied [29]. On the other hand, overt or clinical primary hypothyroidism is defined as TSH concentrations above TSH >10. The American Thyroid Association and the Association of Clinical Endocrinologists guidelines recommend using a TSH concentration of 4.12 mIU/lto classify individuals with subclinical hypothyroidism. However, the recommendation included monitoring patients with initial TSH between 4.5 and 10 mIU/L every 6-12 months before making a decision to initiate treatment [44, 45]. Although TSH concentrations are primarily used for initial screening, a much more comprehensive thyroid profile, including free thyroxine concentrations, are used prior to initiating treatment. FT4 is available to the tissues and is, therefore, the metabolically active fraction. Elevated FT4 levels support the clinical findings of a diagnosis of hyperthyroidism while low FT4 levels coupled with appropriate clinical findings, can establish a diagnosis of hypothyroidism. Free thyroxine is thus used as an indicator of patient thyrometabolic status. Although, there is data on TSH elevation in subclinical hypothyroidism, decreased FT4 is not always observed in subclinical hypothyroidism, similar to what as observed in this study. Although T4 continued to decrease in cases compared to controls, FT4 remained within normal ranges for this age group of patients. Studies have reported that the presence of antithyroid antibodies, particularly TPOAb, as an indicator to enhanced likelihood of progression to overt hypothyroidism. Although a slightly greater number of patients demonstrated presence of TPOAb compared to age-matched controls, we failed to observe statistical significance.

The prevalence of overt hypothyroidism in the general population varies between 0.3% and 3.7% in the US and between 0.2 and 5.3% in Europe [29, 46–49]. A meta-analysis of studies from around the world including US cohorts estimated the prevalence of sub-clinical hypothyroidism at 5.8% [50]. Similarly, a meta-analysis of studies from Europe estimated the prevalence of undiagnosed hypothyroidism, including both mild and overt cases around 5% [51]. Hypothyroidism occurs more frequently in women, in older people (>65 years) and in white individuals, although data on ethnic difference in emerging [36, 46, 52, 53]. Fatigue is reported in patients diagnosed with hypothyroidism in addition to lethargy, cold intolerance, weight gain, constipation, change in voice, and dry skin, although the clinical presentation can vary significantly based on specific etiologies [28]. Although fatigue as a symptom of treatment has been widely reported in breast cancer patients on treatment and post treatment [14-19, 21], to our knowledge, the association of clinically overt or subclinical hypothyroidism in this patient population has not been associated to fatigue and related symptoms observed in this patient population. Compared to a prevalence of 5-6% in the general population, the prevalence of mild of overt hypothyroidism observed in 15.4% of breast cancer patients post treatment with chemotherapy, compared to 8% in age matched controls is significant. The relatively higher rate of subclinical and overt hypothyroidism observed in the control arm compared to 5-6% observed in the general population can be potentially attributed to the age of the population and the predominant racial distribution of white women in this study [36, 46, 52, 53].

Similar to the results published in previous studies, subjects with breast cancer reported significantly greater fatigue symptoms than age-matched controls. The disruption index scores for subjects with breast cancer were significantly higher than controls at baseline and year 1, with the statistical significance diminishing at year 2 (p=0.08). With several pilot and feasibility studies providing early evidence of some efficacy from interventions with physical activity and obesity management to reduce fatigue related symptoms and improve quality of life, [37–40] it is possible that breast cancer survivors are adopting and incorporating these lifestyle changes to manage fatigue with time.

Overall, we observed that an increasing concentration of TSH >4.0 mIU/L and decreasing FT4 in breast cancer patients were associated with worse symptoms of fatigue, after adjusting for age and BMI, with a statistically significant effect on reported fatigue during the past week.

Progressive weight gain has been well documented by our group and others in breast cancer patients during and after chemotherapy [7, 13, 35], as well as in hypothyroidism [29]. While the cause of weight gain in breast cancer patients remains unknown, some proposed explanations for weight gain in breast cancer patients on chemotherapy include increased food intake as a coping mechanism [13, 54] and decreased physical activity. As reported previously by Huntington and others [7, 35, 54], we observed a significant decrease in physical activity as indicated by reduction in weekly hours of employment and purposeful physical activity. Patients who demonstrated a general decrease in activity level since beginning treatment gained significantly more weight than those who reported no change or an increase in activity level [7, 35]. Huntington also attributes weight gain of over 10 lbs. seen in 50% of patients treated with CMF (cyclophosphamide, methotrexate, and fluorouracil) or CMFVP (CMF plus vincristine and prednisone) to a decrease in activity level during chemotherapy [35]. Thus, progressive weight gain, fatigue and lower physical activity are a common symptom cluster observed both in hypothyroidism and in breast cancer patients post-chemotherapy [27, 55]. Interestingly, although our observations demonstrated that the women classified as overweight or obese reported significantly worse fatigue symptoms than those with normal BMI, we failed to observe an interaction effect of BMI with either group or with thyroid function status. Previous studies have reported that higher BMI was associated with significant greater fatigue, including stress-related neuromuscular fatigue development [56, 57]. Similar to these studies, obese and overweight women reported significantly greater fatigue that interfered with subjects' general level of activity and work activity, irrespective of group (cases and controls) or thyroid function status.

Although this was a pilot trial, we identified several limitations. First, we have limited the cases to breast cancer patients receiving AC plus a taxane. Research has clearly shown that duration of treatment, which varies with chemotherapy regimens, can have an impact on side effects. However, restricting eligibility provided us a homogenous group of women and eliminated confounding factors such as effects of different treatment regimens and durations of chemotherapy. Additionally, when subjects were identified with TSH concentrations >4.0, this information was passed on to the primary care physician for further evaluation and referral to endocrinologist for treatment, to reflect the current clinical practice in the community. Thirdly, our study observations included changes in thyroid function, measuring fatigue symptoms and anthropometric measurements from baseline to 24 months. We failed to measure changes in physical activity, cognitive function, or sleep activity- the symptom cluster that has been now been widely associated and reported in this patient population. We [27, 58] and others have observed increase in the occurrence of fatigue and obesity related to the significant changes in serum estradiol and SHBG concentrations, potentially related to breast cancer patients receiving anti-estrogen tamoxifen (TAM) or with the different types of estrogens diethylstilbestrol (DES), ethinyl estradiol (EE), estradiol (E2) and in studies reporting acute as well as chronic adverse effects, including general fatigue, due to the use of ethinylestradiol in postmenopausal patients with heavily pre-treated metastatic breast cancer. In addition, our study failed to measure these steroid hormone levels and collect information on TAM or AI use in this patient population post chemotherapy. Initiating hormonal therapy is currently the standard of care post chemotherapy. Although these were important variables that may have provided additional data on other contributing factors to fatigue in this patient population, our goal was to ultimately evaluate etiology of fatigue that was conducive to and inform the development of early phase interventions in this patient population.

## MATERIALS AND METHODS

### Selection and description of participants

We conducted a prospective, case-control study in the Suncoast Community Clinical Oncology Program (CCOPs) Research Base membership to assess the magnitude of change in thyroid function in breast cancer patients during or as a sequel to chemotherapy, in addition to assessing whether change in thyroid function is associated with the above cancer therapy-related symptoms in 270 breast cancer patients (cases) patients from diagnosis, through chemotherapy and at 12 and 24 months post initiation of chemotherapy, and 280 healthy volunteers (controls), disease-free, age and menopausal status matched controls. The duration of observation for both cases and controls began at start of treatment for cases and end at 24 months from start of treatment.

**Figure 3:**
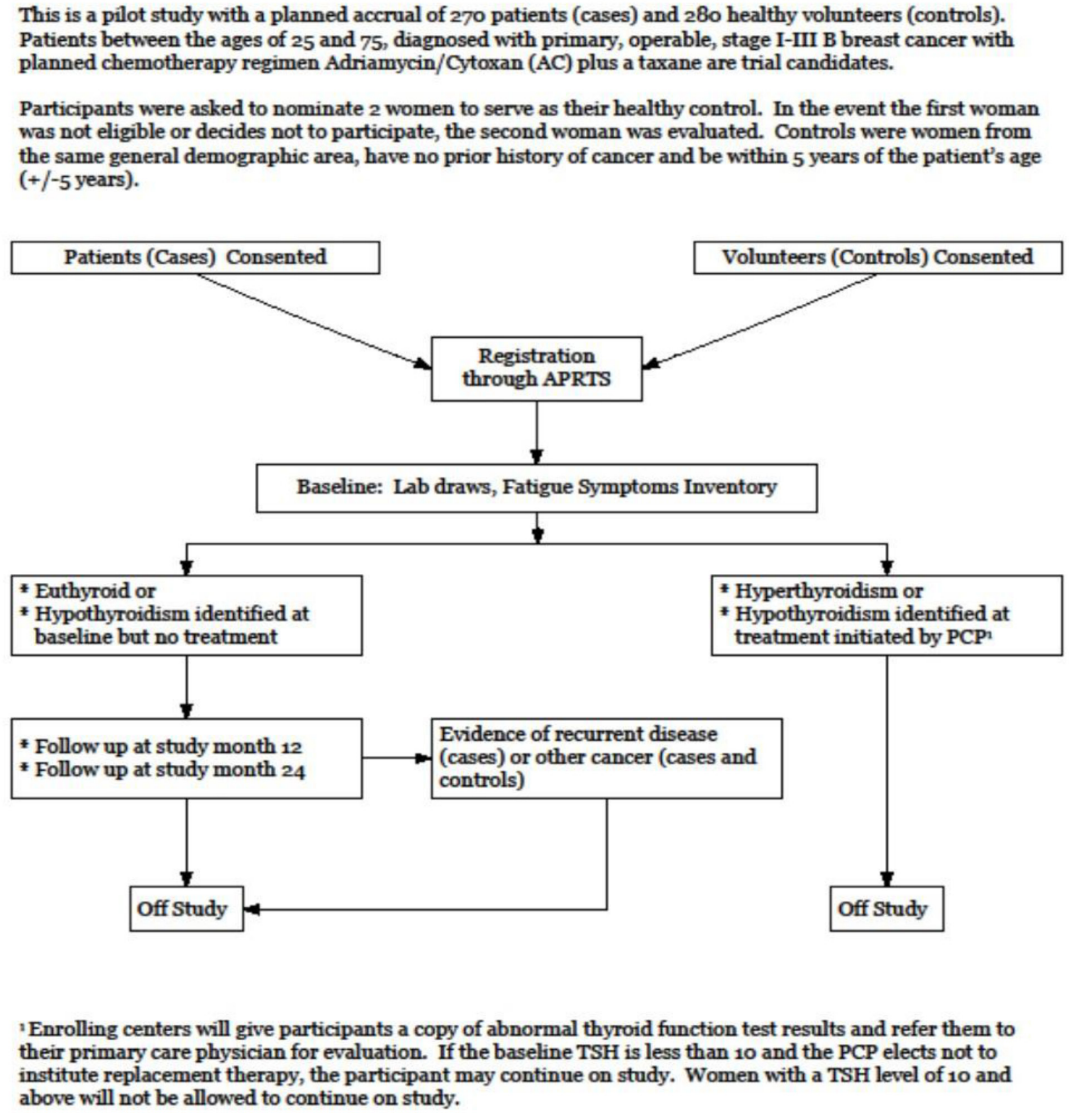
Study schema

Patients between the ages of 25 and 75, diagnosed with primary, operable stage I-III B breast cancer and with planned chemotherapy regimen Adriamycin / Cytoxan (AC) plus a taxane (given with AC or follow AC) were trial candidates. Exclusion criteria included no prior history of other cancers (except non-melanoma skin cancer), stage IV breast cancer, history of adjuvant hormonal therapy or chemotherapy prior to sample collection, other chemotherapeutic regimen other than AC plus a taxane, receiving monoclonal antibodies or other biologic therapy, scheduled to receive Herceptin, previous diagnosis of hyperthyroidism or hypothyroidism and pregnancy or lactation. Cases were asked to nominate 2 women to serve as their healthy control. In the event the first woman was not eligible or decided not to participate, the second woman was included for evaluation. Controls were women from the same general demographic area, with no prior history of cancer and within 5 years of the patient’s age (+/- 5 years), showing no evidence of breast cancer (women ages 40 and older were required to have a mammogram within 2 years of study entry and women under the age of 40 had a clinical breast examination within 2 years of study entry) and willing to have blood drawn at required time points. Women with a history of hyperthyroidism or hypothyroidism and those who were pregnant or lactating were not eligible. Figure 3 shows the study schema. Figure 1 provides a consort diagram. The study was approved by the respective institutional review boards and that all participants signed an informed consent form prior to start of study.

### Technical information

Subjects consented were evaluated at baseline, and at 12 months and 24 months (within 1-2 month window). At baseline we obtained demographic information and history of use of thyroid hormone (for reasons other than thyroid disease) or other steroid hormones. Anthropometric measurements including height, weight and body mass index (BMI) were obtained at baseline, and at 12 and 24 months [27]. Subjects also completed the Fatigue Symptom Inventory (FSI) [59] at each study visit. Finally, non-fasting blood samples (∼20 ml) were collected for markers of thyroid function, including thyroid stimulating hormone (TSH), free thyroxine (Free T4) and thyroid peroxidase antibodies (TPO Ab) at each study visit. All thyroid function marker assays were performed by LabCorp. Subjects who were unable to travel to the enrolling center were allowed to have laboratory specimens drawn at a local LabCorp draw station. Thyroid hormone assays were performed by LabCorp using chemiluminescence assays. If baseline testing results indicated hyperthyroidism or hypothyroidism, the enrolling center communicated test results to participants for follow-up with their primary healthcare provider. Participants with hyperthyroidism or hypothyroidism and a baseline TSH 10 mIU/L or higher were excluded from study. Participants with hypothyroidism who were not started on thyroid hormone continued on study if the TSH concentration was below 10 mIU/L. Those with hypothyroidism who initiated treatment did not continue on study. Similarly, participants who developed hypothyroidism during the study did not continue on-study if replacement therapy was initiated or if the TSH concentration was 10 mIU/L or higher. Patients with evidence of disease recurrence or a secondary primary, and controls who developed cancer did not continue on study. All study participants were paid $50 for each completed study visit. Entry into this study was open to women of all ethnic backgrounds.

### Statistical analysis

Baseline demographic characteristics were compared between women with breast cancer and matched controls by a paired t-test for continuous data and a Cochran-Mantel-Haenszel test for discrete data. McNemar test was used to compare the proportions of women who developed subclinical (TSH>4-10 μIU/mL) or overt hypothyroidism (TSH>10 μIU/mL or higher) between cases and controls. McNemar test was also used to compare the proportions of women with positive TPO Ab at each visit. FT4 measurements were compared between groups by a non-parametric rank method (Wilcoxon Signed Rank test). The ordinal scale of fatigue symptoms was compared between cases and controls at each visit by a Wilcoxon Signed Rank test. Further analyses were done to determine if hypothyroidism had a significant effect on fatigue symptoms in patients diagnosed with breast cancer using a generalized estimating equations (GEE) repeated measures analysis. The GEE models were adjusted by age and BMI. Each symptom was analyzed as a dependent outcome with the identity link between mean response and covariates. The GEE analysis was also used to determine the influence of BMI on fatigue symptoms. Covariates in the GEE included BMI (overweight, obese vs. normal), age, visit, group (cancer cases vs. healthy controls) and thyroid status (hyperthyroidism vs. euthyroidism), and the interactions of BMI with case versus control and BMI with hyperthyroidism vs. euthyroidism.

Statistical analyses were performed with SAS (version 9.4; SAS Institute, Cary, NC). All tests of significance were two tailed. P <0.05 was considered statistically significant. P value was not adjusted for multiple testing, as our a priori intent was to test each variable independently.

## CONCLUSIONS

Our study applied a quick and a cost-effective approach to evaluate the plausible correlation of the prevalence of thyroid dysfunction in this patient population. The goal of this study was to characterize variances in thyroid function, including subclinical abnormalities, in breast cancer patients compared to controls at diagnosis and after chemotherapy. Our study sought to investigate an important problem that needs to be answered definitively. This study allowed us to acquire estimates of the proportion of screened asymptomatic patients with primary hypothyroidism or subclinical hypothyroidism prior to treatment for breast cancer. In addition, it allowed us to obtain estimates of the magnitude of change in thyroid function with chemotherapy in this population compared to an age-matched control group observed for the same period of time. Although fatigue has been identified as a disabling symptom in breast cancer patients post-chemotherapy, to date, there are no interventions –pharmacological or non-pharmacological- that have demonstrated efficacy in ameliorating this symptom and the related cluster of symptoms that have a profound effect on quality of survival in this patient population. Our study provides preliminary data of the effect of decreased thyroid function as it relates to symptoms of fatigue. In addition, it demonstrated that a subgroup of obese women (BMI >30) post-treatment experience more significant fatigue than their leaner counterparts. Screening breast cancer patients for thyroid function status at diagnosis and serially post-chemotherapy is critical. Additionally, recognizing breast cancer patients with baseline BMI >30 will further identify a subgroup at a relatively greater risk for fatigue. Future well-powered randomized clinical trials should be undertaken to evaluate the efficacy and safety of treating patients diagnosed with hypothyroidism, including subclinical disease post- chemotherapy for breast cancer, with thyroid hormone therapy and observe the extent to which symptoms of fatigue, weight gain and lethargy can be reversed.

## Abbreviations

AC: Adriamycin and Cytoxan
BMI: Body Mass Index
CMF: Cyclophosphamide, methotrexate, and fluorouracil
CMFVP: Cyclophosphamide, methotrexate, fluorouracil, vincristine and prednisone
CCOPs: Community Clinical Oncology Programs
CRF: Cancer related fatigue
Free T4: Free thyroxine
FSI: Fatigue Symptom Inventory
GEE: Generalized estimating equations
QoL: Quality of life
REE: Resting Energy Expenditure
SAS: Statistical Analysis System
TPO: Thyroid peroxidase
TSH: Thyroid stimulating hormone
US: United States of America
